# Supplementary material for: Aberrant Methylation of the Imprinted C19MC and MIR371-3 Clusters in Patients with Non-Small Cell Lung Cancer
Source: Cancers (Basel). 2023 Feb 25;15(5):1466. doi: 10.3390/cancers15051466 (PMC10000578; doi:10.3390/cancers15051466)
Supplement: Supplementary file 1 [file cancers-15-01466-s001.zip › cancers-2227400-supplementary/Table S1 20022023.pdf]

Supplementary table S1. Characteristics of the study and control cohorts.

	Patients (N=47)	Controls (N=23)
**Gender**		
Male	76.6 (36)	87.0 (20)
Female	23.4 (11)	13.0 (3)
**Age (years)**	67 [60-73]	35 [21-62]
**Smoking status**		
Smokers	40.4 (19)	30.4 (7)
Ex-smokers	44.7 (21)	21.7 (5)
Non-smoker	14.9 (7)	47.8 (11)
**Packs-year**	41.0 [20.0-65.7]	2.0 [2.0-20.0]
**Histology**		
Lung adenocarcinoma	57.4 (27)	-
Squamous cell carcinoma	42.6 (20)	-
**Staging**		
Stage I	40.5 (19)	-
Stage II	38.3 (18)	-
Stage III-IV	21.2 (10)	-
**Subjects with COPD**	42.6 (20)	17.4 (4)

Continuous variables are expressed as median [interquartile range (IQR)] and categorical variables as number of cases (%).
